# Clinical genetics in cancer prevention

**DOI:** 10.1186/1897-4287-10-S4-A27

**Published:** 2012-12-10

**Authors:** Pavel Elsakov

**Affiliations:** 1 Institute of Oncology Vilnius university, Santariskiu st. 1, Vilnius LT-08660, Lithuania

## 

Cancer family history has been known to be one of the main risk factors. Members of high – risk families should be given recommendations, which may improve prophylaxis, early diagnosis and treatment. At present time is possible to identify several genes involved in the hereditary forms of some types of cancers including colorectal and breast/ovarian. Hereditary forms of breast cancer are mostly caused by mutations in such genes as BRCA1/2 and of hereditary non-polyposis colorectal cancer in genes HLMH1, HMSH2 or HMSH6. Previous studies of Lithuanian population concentrated on breast/ovarian cancer families identified three recurrent mutations 4153delA, 5382insC and C61G in BRCA1 gene. Later studies of unselected cases breast and ovarian cancer patients found that 6% of breast and 19% of ovarian cancer carried a founder mutation in the BRCA1 gene. The majority of mutation-positive patients did not have a significant family history: 71.4 % of the breast cancer patients and 87.5% of the ovarian. These and others clinical genetic aspects raise a necessity to change view on routine cancer prevention strategy, as mammography screening for breast cancer with BRCA1/2 mutations, screening by IFOBT and colonoscopy for HPCC and it is necessary to change the view on combination of chemotherapy (including neo-adjuvant) with BRCA1 positive breast cancer (anthracyclines/taxans).

